# Role of Dendritic Cells in Inflammation

**DOI:** 10.3390/ijms21124432

**Published:** 2020-06-22

**Authors:** Marcello Chieppa

**Affiliations:** National Institute of Gastroenterology “S. de Bellis”, Institute of Research, Via Turi, 27, 70013 Castellana Grotte, Italy; marcello.chieppa@irccsdebellis.it

From the first manuscript describing “A Novel Cell Type in Peripheral Lymphoid Organs of Mice” [1], dendritic cells (DCs) become a major subject of investigation in immunology. Uninterruptedly, for the last 20 years, more than 50.000 studies mentioning DCs were published due to their pivotal role in antigen presentation and initiation of the adaptive immune response.

Throughout this research topic, several aspects of DCs differentiation, maturation, and response to infections were described with the intent of developing strategies to bend DCs’ inflammatory abilities to the needs of disease resolution.

DCs plasticity was noted shortly after their discovery, but one of the first anatomical compartments to be described as able to “educate” DCs’ inflammatory response was the intestine [2]. Mucosal tissues are complex environments characterized by the constant interplay between microbiota and mucosal DCs. The review by Dr. Meghil highlights the cascade of events that may lead from oral loss of tolerance toward chronic inflammation and the onset of several diseases [3].

The tissue microenvironment changes dramatically during pathological conditions and consequently the immune response. Another example of this phenomenon is represented by the interplay between the innate and adaptive immune factors in the atherosclerotic microenvironment described in the review by Dr. Herrero-Fernandez [4].

DCs progenitors migrate into different tissues and mature into different DCs subtypes that likely adapt to the needs of the tissue. Different tissues may imprint different DCs characteristics in response to the abundance of inorganic metals such as iron or calcium. From different perspectives, Dr. Verna and Dr. Naert address the axis between metals abundance and the differentiation and maturation of mouse CD11c+ BMDCs [5,6].

DCs’ maturation is crucial for antigen presentation and cross-presentation of exogenous peptides on major histocompatibility complex class I molecules to prime naïve CD8^+^ T cells response. The review by Dr. Imai presents recent advances in the understanding of the intracellular transport routes for correct antigen cross-presentation [7].

DCs’ maturation is also related with chemokine receptor switch allowing DCs migration toward lymphnodes to present antigens. The article by Dr. Gaffal reports the role of cannabinoid receptor 2 in modulating the maturation phenotype of DCs without affecting their chemotactic capacities using a murine model of contact hypersensitivity [8].

Viral infections are associated with increased incidence of severe sepsis. The article by Dr. Howe identifies in the role of IFNβ and type I IFN receptor in mice susceptibility to sepsis using WT versus KO mice [9].

Latent viral infection may also characterize multiple sclerosis patients as proposed in the communication by Dr. Corsetti in light of the enhanced activation of plasmacytoid dendritic cells from multiple sclerosis patients [10].

Overall, this research topic highlights several aspects affecting DCs’ maturation, activation, migration, and antigen presentation. Each one of these aspects is tightly regulated by a complex cascade of transcriptional factors and post-transcriptional regulators, which are known as microRNAs. The review by Dr. Scalavino sheds light on the importance of miRNAs in inflammatory processes mediated by DCs in physiological and pathological conditions and to the potential translational application for future therapies [11].
